# Annotations

**Published:** 1893-03-04

**Authors:** 


### Annotations.

**The Doctor Day by Day.**

The *Practitioner* for February opens with a lecture by Dr. Lauder Brunton, which admirably illustrates our unceasing contention that physic must be brought down from the clouds and carried to the actual bedsides of all sorts and conditions of men. Dr. Brunton expounds the "action of drugs on the bladder and genital organs." Now the stupidest doctor in Great Britain, if there be any stupid ones, will not charge Dr. Brunton with being "a merely practical man." On the contrary, he is scientific to his very finger tips: he exhales science from every pore of his body. And yet his lecture is a perfect model of common sense and practicality. It illustrates another of our standing contentions, that it is only the truly scientific man who can be truly and in the highest sense practical. Wherein—let us ask ourselves—wherein consists the essence of Dr. Brunton's admirable practicality. It consists in these several circumstances: That he knows the normal structure and functions of the human body and of all its parts; that he knows the abnormalities—that is, the diseases—as well as the normalities; that he understands the nature and action of the tools of his trade, that is, drugs and other hygienic factors; and that he has a sincere desire to use his knowledge for the benefit of his patient. This is the perfection of science, and it is so simple that any man of fairly good intellect and of patient industry may in due time become as scientific and as practical as Dr. Lauder Brunton. The *Practitioner*, as we have stated before, is one of the ablest and most useful of all monthlies. As a combination of science, sense, simplicity, and practice it has few equals.

**Strength.**

It used to be often objected against Carlyle that he worshipped mere "strength"—"brute force." Physiology, however, would have sided with Carlyle and against the objectors. "To him that hath shall be given," says physiology, "and from him that hath not shall be taken away even that which he hath." This is a hard saying, but is it also a true one? Most certainly it is! Among all the things which are valuable in the world mere strength ranks with the highest. It is what philosophers would call an "absolute good." Many people kick against this doctrine. They are merely kicking against the pricks, and are fools for their pains. Strength is one of those things which everybody should strive to acquire. It is capital—capital which can be put to an almost greater variety of uses than any other form of capital. Of all the forms of strength moral strength is the highest, the most valuable, the most enduring. For its full and perfect development moral strength needs mental and this again needs physical. Strength when combined in this somewhat rare trinity is invulnerable. Of all animal forms the vertebrate, the backboned are the most striking illustrations of strength, and of vertebrate animals those alone which display the trinity of moral, mental, and physical strength are the upright backboned, the creatures we call "human." This is full of significance for the individual and for the mass. In states the small and the struggling often show the firmest backbone—the most strength in its three orders. Larger states tend to revert to an invertebrate condition. That is a fatal condition for states as for individuals. The most urgent of patriotic duties for men in a vast and wide-spreading state like our own at the present moment is the stiffening of the backbone. The man who stiffens his backbone need not, therefore, shut his eyes, neither need the state. We must open our eyes, but we must certainly stiffen our backbones. The state is an organism, as Mr. Herbert Spencer has shown us. It must constantly go back upon first principles, and nowhere are first principles seen in such convincing object lessons as in physiology. Strength, says physiology, is the parent of all good things—strength physical as the root, strength mental and moral as the branches and fruit. Give us but the trinity of strength in the individual and the state and all will be well.

**Influenza once More.**

The question whether we are or are not to be again visited by influenza seems to be answered beyond doubt. The enemy is upon us already, and his attacks are increasing in strength and frequency. We have learnt to respect our foe by this time, and to meet him with a due sense of apprehension mingled with intelligent resolution. It is true we have not yet settled the question as to the particular microbe or family of microbes to which we are indebted for the visitation. But we have settled a question which is much more practically important: the question of successful treatment. As is so often the case in medicine, a "specific" is not yet found—probably it never will be. But for all that, no intelligent physician is in the least doubt as to what ought to be done. The treatment resolves itself into three main principles: the unloading, in the first place, of the *primæ viæ* and the consequent general diminution of vascular tension; the most careful protection against chills; and the judicious feeding with highly nutritious but easily digestible food. These are sound principles of treatment, and they lead to entirely satisfactory results. On two points the public may be consoled: the disease does not appear to be either so severe or so contagious as in the two previous epidemics. A considerable number of cases have come under the writer's own notice which, whilst they have undoubtedly been of the type of *La Grippe*, have not been either excessively painful or excessively prolonged. The sequelæ, too, have been less urgent and dangerous. Nevertheless, whilst we desire to allay apprehension, we counsel in the event of an attack, prompt care, rest in bed, and competent medical treatment until strength is completely restored. In speaking of influenza, it would be well to remember that former outbreaks of this disease have been followed by epidemics of cholera. It has been specially noted that influenza, after prevailing two or three years, has given place to very serious cholera epidemics. No class of men can be more anxious to prevent an epidemic of cholera than medical men. But doctors are comparatively powerless if the public will not help itself. All intelligent persons should take every opportunity of learning the main principles of sanitation; but above all they should put what they know into practice, whether the knowledge be little or much.

